# Video Speed Switching of Plasmonic Structural Colors with High Contrast and Superior Lifetime

**DOI:** 10.1002/adma.202103217

**Published:** 2021-08-26

**Authors:** Kunli Xiong, Oliver Olsson, Justas Svirelis, Chonnipa Palasingh, Jeremy Baumberg, Andreas Dahlin

**Affiliations:** ^1^ NanoPhotonics Centre Cavendish Laboratory University of Cambridge Cambridge CB3 0HE UK; ^2^ Department of Chemistry and Chemical Engineering Chalmers University of Technology Gothenburg 41296 Sweden

**Keywords:** conductive polymers, electronic paper, structural colors, video

## Abstract

Reflective displays or “electronic paper” technologies provide a solution to the high energy consumption of emissive displays by simply utilizing ambient light. However, it has proven challenging to develop electronic paper with competitive image quality and video speed capabilities. Here, the first technology that provides video speed switching of structural colors with high contrast over the whole visible is shown. Importantly, this is achieved with a broadband‐absorbing polarization‐insensitive electrochromic polymer instead of liquid crystals, which makes it possible to maintain high reflectivity. It is shown that promoting electrophoretic ion transport (drift motion) improves the switch speed. In combination with new nanostructures that have high surface curvature, this enables video speed switching (20 ms) at high contrast (50% reflectivity change). A detailed analysis of the optical signal during switching shows that the polaron formation starts to obey first order reaction kinetics in the video speed regime. Additionally, the system still operates at ultralow power consumption during video speed switching (<1 mW cm^−2^) and has negligible power consumption (<1 µW cm^−2^) in bistability mode. Finally, the fast switching increases device lifetime to at least 10^7^ cycles, an order of magnitude more than state‐of‐the‐art.

## Graphical abstract

Electronic paper technologies are essential for reducing the energy consumption in future display devices. The first example of video speed switching of structural colors over the whole visible region is shown, while retaining high contrast. This is achieved with polarization‐insensitive metasurfaces and conjugated polymers, not liquid crystals. The lifetime is shown to be over ten million cycles.

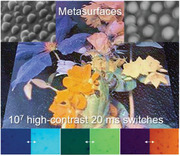

## 1 Introduction

Electronic displays are becoming increasingly common for communication, advertisement, active signage, and visual entertainment. Ordinary emissive displays based on light emitting diodes (LED) or liquid crystals (LCD) can provide excellent color image quality and video display, but at the cost of considerable energy use. There is little room for further improving the electricity‐to‐light energy conversion and the power consumption becomes especially high when visibility in daylight is necessary. A solution to this problematic energy wastage is to use reflective displays, also known as “electronic paper,” which simply reflects environmental light. This leads to extremely low power consumption,^[^ ^1^ ^]^ improved visibility in bright environments and potentially health benefits.^[^ ^2^ ^]^ Recently, a new direction of research has emerged focusing on active control of plasmonic structural colors^[^ ^1^, ^3^ ^]^ and electronic paper is one important application in this field.

However, whether using plasmonic nanostructures or not, it has proven very difficult to develop electronic paper with a performance comparable to emissive displays.^[^ ^4^ ^]^ Widespread commercial devices are based on electrophoretic ink^[^ ^5^ ^]^ (Amazon Kindle etc.) and suffer from poor image quality in color mode, which is achieved by subpixels containing red, green, and blue (RGB) filters.^[^ ^6^ ^]^ In addition, the slow switching (≈1 s) prevents video playback and limits the usage to applications such as e‐readers and simple labels. The electrowetting technology appeared as an important electronic paper technology because it provides video speed,^[^ ^7^ ^]^ but it remains unavailable commercially. The human eye perceives refresh rates of >20 Hz as motion pictures and flickering disappears entirely at ≈50 Hz. Such fast refresh rates can be achieved with LCD displays, but the image visibility becomes poor in the reflective configuration^[^ ^8^ ^]^ (<15% absolute reflectivity). Organic and inorganic electrochromic materials have emerged as strong candidates for high contrast polarization‐independent switching over the visible spectral region,^[^ ^9^ ^]^ but their response time is normally too slow for video display (hundreds of ms or even more for transition metal oxides). It is generally believed that although structural colors are highly interesting for electrochromic devices, the switching cannot be made fast enough for video applications, especially not if the contrast should be high (≈50% change in absolute reflectivity or transmittance). For conductive polymers, limitations in switch speed have mainly been attributed to the relatively slow “diffusion” of ions in the electrolyte and the polymer film during the doping process.^[^ ^10^ ^]^ A few exceptions exist, such as polyaniline, which is known to change protonation state very fast.^[^ ^11^ ^]^ It is also known that extremely thin films of the commonly used polymer poly(3,4‐ethylenedioxythiophene) (PEDOT), can switch at video speed.^[^ ^12^ ^]^ Unfortunately, this has little relevance for electrochromic devices because the film must be considerably thicker in order to reach good contrast.^[^ ^13^ ^]^ One way to increase contrast is to prepare thin PEDOT films on wires^[^ ^14^ ^]^ or tubules^[^ ^12b^ ^]^ with high surface area, but such structures are not useful for structural coloration and complex to prepare. Furthermore, the polymers mentioned above switch between different coloration states,^[^ ^15^ ^]^ and are less suitable for broadband absorption modulation.

In this work we show high‐contrast broadband‐switching of plasmonic structural colors at video speed. Our metasurfaces are easily prepared over large areas in a scalable process and provide a complete color range. We systematically investigate which factors influence switching speed and illustrate the importance of active transport of ions in the electrolyte, i.e., ion drift in a field. In combination with the increased area and curvature of the nanostructures this enables excellent switching performance at very high refresh rates. Furthermore, it is shown that as the system approaches the fast‐switching regime, the optical response starts to obey first order reaction kinetics. In addition, superior device lifetime (>10^7^ switches) is achieved as a consequence of the short pulse durations. Finally, the power consumption is shown to be ultralow at video speed operation and the devices support bistability (coloration memory), which leads to practically zero power usage for static images.

## 2 Results and Discussion

**Figure 1:**
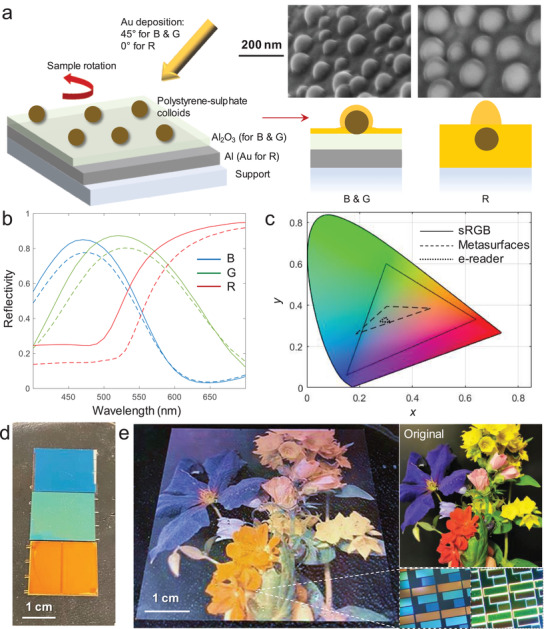
Plasmonic metasurfaces with nanoscale protrusions. a) Fabrication by colloidal encapsulation and electron microscopy images. b) Reflectivity spectra of red, green, and blue surfaces in air (solid lines) or with a thin layer of water (dashed lines) measured under diffuse illumination. c) CIE chromaticity coordinates in the electrolyte environment. For comparison, the standard RGB gamut and the gamut of the latest commercial color e‐reader are shown. d) Photo of large‐area primary‐colored samples immersed in water. e) Color image reproduction with pixelated metasurfaces. (The original image is also shown.) The magnifications show RGB subpixels under bright reflective (left) or dark field (right) illumination.

We prepared new plasmonic metasurfaces by combining established nanofabrication techniques. Physical deposition of gold was performed onto a short‐range ordered colloidal sphere monolayer on Al and Al_2_O_3_ (**Figure** **1**a). In contrast to previous work,^[^ ^13^, ^16^ ^]^ we here use tilting and rotation to create an ultrathin (12 nm) conformal gold coating over the spheres, which are then left buried on the surfaces.^[^ ^17^ ^]^ Reflectivity spectra were measured under diffuse illumination with a standardized colorimetric tester,^[^ ^13a^ ^]^ showing high polarization‐independent reflectivity (Figure 1b) and a wide RGB gamut in the CIE *xy* diagram. For comparison, we also include the color gamut of the latest commercial electronic reader in color^[^ ^6^ ^]^ (PocketBook with E‐Ink Kaleido released in 2020), which showed much poorer color quality (Figure 1c). The blue and green metasurfaces provide structural coloration based on a Fabry–Pérot cavity resonance and the plasmonic activity of the dielectric voids inside the gold.^[^ ^17^, ^18^ ^]^ Numerical simulations with literature values for metal permittivity^[^ ^19^ ^]^ showed excellent agreement with experimental spectra and the near field plots confirmed the contribution of localized resonances (Figure S1, Supporting Information). The Al_2_O_3_ thickness was used to tune the color in the blue‐green region (83 nm for blue, 98 nm for green). Red surfaces were created by depositing a thicker (80 nm) gold layer at normal incidence, using a single gold layer as the underlying mirror and no dielectric cavity. The coloration of the red metasurfaces originates from plasmon resonances due to the “bumps” on the surface (Figure S1, Supporting Information) and to some extent inherent absorption of blue light by gold. The structural colors were highly insensitive to the viewing angle (Video S1, Supporting Information). Other colors could be achieved by tuning structural parameters and the metasurfaces were possible to fabricate on flexible supports (Figure S2, Supporting Information). For the rest of this paper we focus on results for RGB metasurfaces on glass (photo in Figure 1d). Note that the width of the reflectivity peaks shows a good balance between chromaticity and reflectivity: more narrow reflection peaks will certainly improve color quality but will also strongly reduce the overall visibility under broadband illumination. (We recently showed how this effect can be illustrated by the *Y* parameter.^[^ ^6^ ^]^)

In order to illustrate what a color image would look like when viewed in a reflective display device based on the metasurfaces, we took a photo of flowers (Image S1, Supporting Information) and patterned the corresponding pixels as RGB triplets by three photolithography steps^[^ ^13b^ ^]^ (Figure 1e). The subpixels scattered their complementary colors under dark field illumination (see also spectra in Figure S3, Supporting Information), thereby verifying the plasmonic activity. However, the scattering was very weak and most of the light which is coupled to plasmons becomes resonantly absorbed by the metal, as expected for relatively small particles/voids^[^ ^20^ ^]^ (we used 50 nm colloids). We emphasize that although our new structures show high reflectivity and chromaticity, they are still comparable to previously presented metasurfaces in this respect.^[^ ^13^, ^16^ ^]^ Instead, the main point of the nanostructures produced in this work is their surface structure. Besides providing structural colors, the nanostructure increases the active area (compared to a planar surface) by up to 4*πΓR* ^2^, where *R* is the radius of the particles and *Γ* is their coverage (≈70 µm^−2^). Note that besides increasing the area, the nanoscale protrusions also create a high positive surface curvature. We hypothesized that this could provide enhanced contrast for a thin electrochromic film grown on the metasurfaces and that such a film could switch with video speed under the right conditions.

To systematically investigate what influences the switch speed for a conjugated polymer, which becomes doped by ions, we first electropolymerized dimethylpropylenedioxythiophene (PProDOTMe_2_). The details of the polymerization have been described previously.^[^ ^13a^ ^]^ Among all conjugated polymers that can be prepared by electropolymerization, PProDOTMe_2_ is established as ideal for high‐contrast broadband (black/transparent) switching.^[^ ^21^ ^]^ We continued under the assumption that the switch speed is to a high extent determined by the transport of ions in the solvent as well as inside the polymer film network.^[^ ^10^ ^]^ PProDOTMe_2_ is absorbing in its native state and becomes transparent when the monomers are oxidized at anodic potentials and anions enter the film.^[^ ^22^ ^]^ Given that there are not enough ions in direct contact with the polymer to reach the full doping capacity and complete the switch, some need to be transported to the polymer film (and also move within it). We hypothesized that this movement is not simply free diffusion because the ions are exposed to forces from the electric field generated when the potential is applied (or when its polarity is reversed). Under the assumption that the ions are accelerated instantly, their velocity can be estimated by an electrophoretic force balanced by Stokes friction

(1) $$\begin{array}{c} v=\frac{zeE}{6\pi \eta R} \end{array}$$

In this model, *v* depends on the hydrodynamic radius *R* of the ions, the solvent viscosity η and the field strength *E* (*z* is valency and *e* the elementary charge). Although Brownian motion also occurs, Equation (1) gives the average velocity along the direction of the field (strictly *E* and *v* are vectors). The choice of anion may influence the speed, but we mostly used non‐aqueous solvents with LiClO_4_ since such electrolytes are stable and compatible with the materials in the nanostructures.^[^ ^13a^ ^]^ Similarly, we noted that increasing the voltage *U* used for switching clearly enhanced the switch speed, in agreement with an enhanced electrophoretic mobility, but this parameter is quite straightforward to optimize: The voltages should simply be as high as possible in magnitude to increase doping capacity while still avoiding damage to the polymer and the supporting structure.^[^ ^13a^ ^]^ We generally used −1.0 and +0.5 V versus Ag/AgCl for switching the polymer between its transparent and absorbing states.

**Figure 2:**
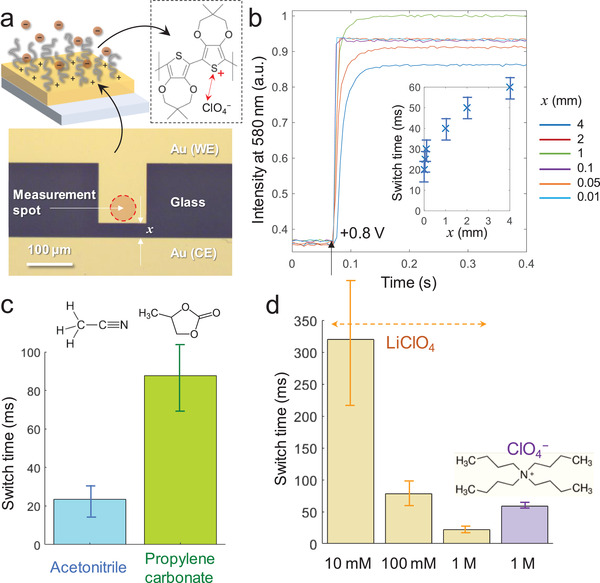
Investigating what influences switch speed using planar gold. a) Example of a gold micropattern with controlled lateral distance to the counter electrode. b) Intensity time trace during a switch (1 m LiClO_4_ in acetonitrile) from −0.7 to +0.8 V (vs Au) with different distance to the counter‐electrode. The small intensity variations are due to a not entirely uniform polymer film over distances of several mm. (Note that these intensity variations do not correlate with speed.) The inset shows the corresponding switch times (>95% of intensity change). c) Switch time in propylene carbonate in comparison with acetonitrile (1 m LiClO_4_, electrode separation 100 µm). d) Switch time for different electrolytes (acetonitrile, electrode separation 100 µm). Error bars are four standard deviations.

To evaluate the role of field‐driven ion migration, we reduced the distance *x* between electrodes in “horizontal” two‐electrode device designs,^[^ ^23^ ^]^ thereby increasing *E* = *U*/*x*. We initially used planar gold electrodes and effects from the nanostructure were investigated separately (as described below). Importantly, the lateral electrodes allow an independent investigation of the role of the field strength since all other parameters are kept constant. Laser lithography was used to prepare a set of microelectrodes with highly precise control of *x* (example in **Figure** **2**a). Indeed, reducing *x* to the microscale strongly improved the switch speed while keeping all other parameters constant (Figure 2b), as revealed from high temporal resolution spectroscopy.^[^ ^24^ ^]^ Herein, we define the switch speed as the time needed to reach >95% of the intensity change. We show intensity time traces at 580 nm where the contrast for PProDOTMe_2_ is highest.^[^ ^21^ ^]^ The kinetics were very similar at other wavelengths (Figure S4, Supporting Information) and the contrast is similar within the spectral region where the human eye is sensitive.^[^ ^25^ ^]^ We also noted that faster switching was achieved when the optical measurement was performed closer to the edge of the electrode, while the speed was reduced further away from the lateral “gap” (Figure S5, Supporting Information). Importantly, this is not due to effects from limited lateral conductivity^[^ ^26^ ^]^ since these systems have metallic conductors as support. (Also, the dependence should then be the opposite of what we observed.) There is no significant potential drop in the electrode itself, as is often observed in non‐metallic lateral devices.^[^ ^12^, ^27^ ^]^ Still, in the horizontal configuration, the field strength just at the metal surface is obviously reduced when moving along the electrode (simulation in Figure S6, Supporting Information) and this is the reason for the “switching front.” As confirmation, in a vertical cell configuration (parallel electrodes and sandwiched electrolyte^[^ ^23^ ^]^), which gives a homogenous field, the switch process appeared entirely uniform. Throughout this study we tested many different electrode size and shapes, comparable to the pixels used for image reproduction (Figure 1e). We noted no effect on the switch speed as long as the distance to the other electrode was kept constant (example in Figure S7, Supporting Information) and given that the counter electrode had larger charge storage capacity.

In the lateral devices, both gold electrodes contained the polymer, so that ion doping always occurred at one electrode or the other to complete the circuit. As a result, we observed that the “on” switch, where anions need to enter and move through the polymer layer, was slower when the electrode where the reflectivity was measured had a thicker polymer layer. (Both electrodes had the same area.) Conversely, the “off” switch was slower when the working electrode had a thinner layer. When using a three‐electrode configuration with a large area Pt counter electrode, the on switch was slower than the off switch. This confirms that the ion movement is an important bottleneck for speed because when switching off, the counterions are “kicked out” from the polymer film and do not need to move into it. However, we emphasize that this was the behavior observed for the particular voltages used. For instance, for a less negative voltage (e.g., −0.5 V vs Ag/AgCl) and the same positive voltage (+0.5 V vs Ag/AgCl), the off switch was slower. This can be attributed to changes in polymer conductivity with oxidation state.^[^ ^28^ ^]^

We also compared switching with LiClO_4_ in propylene carbonate and acetonitrile solvents, which differ considerably in viscosity.^[^ ^29^ ^]^ In acetonitrile the switch speed was around a factor of four shorter (Figure 2c). This is consistent with a reduced drag force, but other effects should not be excluded (e.g., changes in polymer morphology or ion solvation shell).^[^ ^30^ ^]^ We also verified that reducing the ionic strength decreased the switch speed considerably (Figure 2d), which illustrates how ions must be transported a longer distance on average when they are fewer. In addition, a speed reduction was observed when using the large tetrabutylammonium (TBA) cation, suggesting that removal of like‐charged species from the polymer film also is important for the oxidation process to proceed.

Our results, in particular those in Figure 2, confirm the importance of ion transport on switch speed, which is often mentioned.^[^ ^10^ ^]^ However, the ion transport is normally referred to as purely diffusive in existing literature on electrochromic devices.^[^ ^12^, ^15^, ^30^, ^31^ ^]^ This ignores the fact that there is an electric field present from the moment when the potential is altered. We do not see how our findings (e.g., Figure 2b) can be explained under the assumption that the field, although transient, has no influence on the ion movement. Furthermore, we do not see why ion drift motion should be disregarded as is an established fact (Nernst–Planck equation). We note that a few studies have actually investigated field‐driven ion motion, although inside the polymer film and over relatively long lateral distances.^[^ ^27^, ^32^ ^]^ Also, the importance of drift motion for the injection of ions into a polymer film has been discussed in theoretical work.^[^ ^27^ ^]^ Interestingly, in the closely related research area of organic electrochemical transistors, it is well known that the gate needs to be positioned close to the source and drain to enhance transistor speed and ion drift in the electrolyte is sometimes considered in the modelling.^[^ ^33^ ^]^ In summary, we do not see our findings as controversial, but there is clearly no consensus on the importance of the electric field for switch speed. Hence, we see a need to communicate our results, which should also be of great practical importance for enhancing the switch speed in all the electro‐optical devices which are currently being developed.^[^ ^1^, ^,3a,b,d^ ^]^

Our results so far show that fast switching, compatible with high frame rate video, is possible by selecting the right electrolyte and an electrode configuration with small separation distances. However, for the data in Figure 2, the PProDOTMe_2_ thickness was kept quite thin (≈10 nm). This is considerably less than the amount expected to give best contrast, which can be up to ≈100 nm or more depending on wavelength.^[^ ^13a^ ^]^ Furthermore, only planar gold electrodes were used. In order to increase contrast and to investigate the role of the nanostructure,^[^ ^15^ ^]^ we measured the switch speed for planar gold and metasurfaces for different thickness of PProDOTMe_2_. For the rest of this paper, we show results for 1 m LiClO_4_ in acetonitrile unless otherwise stated, with electrode separation distances of ≈100 µm. This can easily be achieved in simple vertical electrochromic cells, suitable for real devices.^[^ ^23^ ^]^

**Figure 3:**
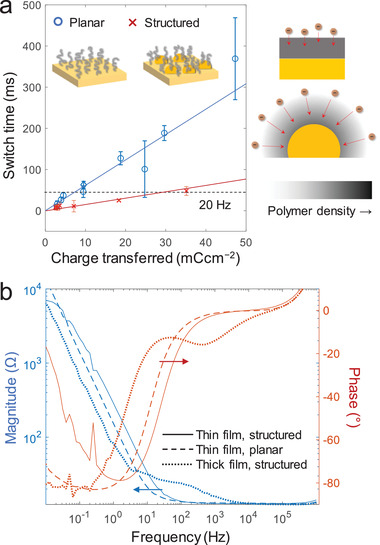
Comparing switching dynamics on planar gold and metasurfaces. a) Switching “on” time (to reach >95% of the full intensity change) versus polymer thickness, represented by charge transferred during electropolymerization growth. Comparison for planar Au and nanostructures (red metasurface). Error bars represent four standard deviations. The schematics illustrate how ion insertion becomes easier for positive surface curvature. b) Impedance spectra for three different cases: thin (≈10 mC cm^−2^) polymer film on a metasurface, thin polymer on planar gold and thick (≈100 mC cm^−2^) polymer on a metasurface.

**Figure** **3**a shows that the switch speed is drastically increased for the nanostructure compared to the planar surface. The polymer thickness is represented by the charge transfer during electropolymerization, where 1 mC cm^−2^ corresponds to ≈8 nm dry thickness.^[^ ^13a^ ^]^ For the same polymer amount, the speed differs by almost a factor of four. This is to some extent explained by the fact that the same polymer amount is spread out over a larger area,^[^ ^12^, ^14^ ^]^ here almost a factor of two more due to the protrusions. In addition, the ion mobility into the polymer is likely further enhanced by the positive surface curvature, which makes the layer more porous (schematics in Figure 3a). This interpretation is in general agreement with early models describing how polymer film morphology changes influence the switch speed.^[^ ^30^ ^]^ During the electropolymerization process, it is expected that most of the polymer forms at positive curvature regions, considering the mass transport of the monomer. Atomic force microscopy images supported this view (Figure S8, Supporting Information). To further verify the importance of surface curvature, we measured switch speeds on samples with less extreme curvature, which indeed gave values in between those of planar surfaces and the metasurfaces (Figure S9, Supporting Information). It should also be kept in mind that the altered electrode geometry influences ion diffusion, i.e., an enhancement effect from the nanostructure might appear even if the ion movement would be purely diffusive.^[^ ^31^ ^]^ However, both the planar surfaces and the metasurfaces show a linear relation between switch time and polymer thickness, which contradicts the quadratic dependence predicted by free diffusion.^[^ ^12^, ^15^ ^]^ This further confirms that ion drift motion also needs to be considered.

Voltammetry sweeps showed no major difference between red metasurfaces and planar gold (Figure S10, Supporting Information). To investigate the dynamics of the circuit, we measured the impedance spectra of both planar electrodes and metasurfaces (Bode plots in Figure 3b). The polymer alters the impedance of the interface at lower frequencies where the capacitive region appears (phase approaching −90°). The capacitive frequency interval shifted to higher frequencies for the metasurfaces compared to planar gold (maintaining the same polymer amount on the electrode). For comparison, a very thick polymer film (≈80 nm) shifted the capacitive behavior to much lower frequencies. Blue and green metasurfaces behaved identically to the red ones to the extent our experimental precision could measure, as expected when the surface structure (particle density, height and diameter) is similar (Figure S11, Supporting Information).

**Figure 4:**
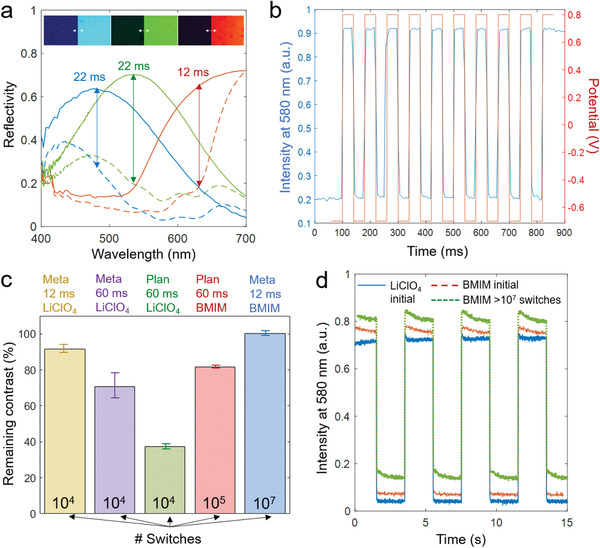
Video speed high‐contrast switching and lifetime analysis. a) Reflectivity spectra during video speed switching. Arrows indicate contrast for red, green and blue. (The red sample has slightly thinner polymer layer and is therefore even faster than blue and green.) The inset shows microscopy image frames for on and off states. b) Multiple full contrast switches (square wave) at video refresh rate, showing voltage and intensity versus time. c) Lifetime analysis measured as relative loss of initial contrast. The number of switches, type of surface, type of electrolyte and pulse duration are altered (LiClO_4_ refers to 1 m of this salt in acetonitrile, “Meta” means metasurface and “Plan” means planar gold). The polymer film thickness was always in the range that gives optimal contrast. d) Intensity trace when switching the same polymer film using LiClO_4_ in acetonitrile or BMIM. For BMIM, the curve obtained after 10^7^ fast switches is also shown. (The curves are shifted vertically for clarity.)

From the data in Figure 3a, it is clear that on planar gold, video speed is only possible for polymer coverages up to a few mC cm^−2^, which is too thin for good contrast.^[^ ^13a^ ^]^ On the metasurfaces, video speed is possible for polymer coverages corresponding to 10–30 mC cm^−2^ depending on the desired refresh rate. This corresponds well to a polymer amount that gives optimal contrast for PProDOe_2_ on a mirror^[^ ^13a^ ^]^ (although this is slightly dependent on wavelength). Indeed, spectra of metasurfaces during video speed operation (≈20 ms switch time) showed excellent contrast (**Figure** **4**a), practically as high as in an optimized system^[^ ^13a^ ^]^ (<10% difference). The spectral changes originate essentially only from the polymer switching, with contributions from surface effects^[^ ^24^ ^]^ found to be negligible (Figure S12, Supporting Information). To rule out any hidden latency effects, we also tested multiple fast switches at video rate. The intensity cycles were fully reproducible over multiple switches for square wave reversal of voltage in a full contrast switch every 40 ms (Figure 4b). We emphasize that this results from the combination of promoting ion drift and introducing positive surface curvature: each of these effects alone did not provide high contrast video speed operation. An example of a fast‐switching metasurface in a liquid cell is available (Video S2, Supporting Information).

Another important benchmark parameter is lifetime, which is a known issue for devices containing organic components. Video speed operation means that the device must perform many switches, which could increase the risk of polymer degradation or release from the surface. Remarkably, we found that operation at video speed improved the device lifetime in terms of number of cycles because of the brief duration of the potential pulses (Figure 4c). While a considerable contrast loss was observed after 10^4^ switches when the potential was applied for 60 ms (longer than what is necessary), the detrimental effect becomes minimal (a few percent) when operating at video speed. Most likely, the short pulse duration limits the occurrence of unwanted side reactions, thereby preventing polymer degradation or release from the surface. Furthermore, using an ionic liquid consisting of the cation 1‐butyl‐3‐methyl imidazolium (BMIM) and BF_4_^−^, we found no loss in contrast (or speed) even after ten million switches, which is an order of magnitude better than previous reports.^[^ ^34^ ^]^ Again, the reason is the short pulse duration, i.e., it is the video speed that provides the long lifetime. It was sufficient to go to a pulse duration of ≈50 ms or more to note substantial degradation, eventually even when using the ionic liquid (Figure 4c). In most devices to date, which do not operate at the frequencies we present here, the pulse duration is by necessity longer in order to complete the switch and this leads to degradation. Changing to the ionic liquid (instead of LiClO_4_) when keeping all other parameters constant gave the same contrast (Figure 4d) and the switch time only increased by a few ms, still compatible with refresh rates up to at least 40 Hz. Note that although the ionic liquid has higher viscosity, it is not surprising that it provides fairly high speed as it consists of only ions (consider the data in Figure 2c,d). Even if some degradation would be observed when approaching hundred million switches, which we have not yet tested for practical reasons, additional lifetime improvements are likely possible. For instance, one can prepare and encapsulate devices in zero humidity environments (no such measures taken in this work).

**Figure 5:**
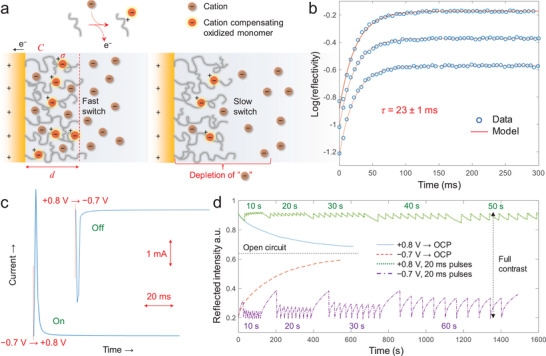
Kinetic analysis and power consumption. a) Illustration of the model of a reaction limited electrochromic switch (left) where the ion transport is much faster than polaron formation. For a slower switch (right), a zone depleted of free ions builds up at the surface. b) Examples of single exponential fits (*R* ^2^ > 0.997) to the logarithm of the reflected intensity during “on” switching (data series are offset for clarity). c) Current time traces during fast “on” and “off” switches (>95% intensity change in 20 ms). Electrode area is 0.8 cm^2^. Note the arrows showing current and time scales. d) Bistability test: applying voltages representing full contrast and releasing (at time zero) to open circuit potential (OCP) makes the intensity slowly approach an equilibrium value (blue and red lines). Brief (20 ms) voltage pulses are sufficient to maintain essentially constant intensity levels (green and violet lines). The time values show OCP durations between the pulses.

We continue with an analysis of the kinetics of the switching process. Presumably, when ions are transported sufficiently fast, another process will become the new speed bottleneck. We propose that this should be the monomer oxidation process (**Figure** **5**a). The doping processes of conductive polymers are complex and not entirely understood.^[^ ^35^ ^]^ Chemical formulae involving monomers and ions are often presented, which implies a mechanism where the ion encounters and reacts with the monomer. At the same time, recent theoretical work suggests that although the counterions are immobile they are simply compensating the charges on the monomers without any chemical interactions,^[^ ^36^ ^],^ i.e., a “3D Stern layer.” This suggests that polaron formation might occur without any counterion present and that ions are attracted to the polymer after it has gained charge. However, even if this is the case, it is clear that further doping will be hampered unless counterions are efficiently transported in to (and like‐charged ions out of) the film to get closer to charge neutrality.^[^ ^35^ ^]^ To further elucidate the mechanism, we analyzed the optical response, which should be a direct measure of the doping progression, using measurements at high resolution with respect to both intensity and time.^[^ ^24^ ^]^

We first derive a model under the assumption that ion transport has been enhanced to the extent that it is fast enough not to limit polaron formation. It is known that the optical absorption of PProDOTMe_2_ films follow the Lambert–Beer formalism fairly well,^[^ ^13a^ ^]^ so the reflected intensity *I* can be written as

(2) $$\begin{array}{c} I=I_{0}R_{0}\exp\left(-2C\sigma d\right) \end{array}$$

where *I*_0_ is the incident intensity, *R*_0_ is the reflectivity of the surface, *C* is the concentration of absorbing entities (polarons), σ is their effective optical cross‐section area and *d* is the polymer thickness (Figure 5a). Ignoring the ions, we postulate that the reaction rate should be proportional to the concentration of monomers available to be oxidized, so that first order kinetics applies

(3) $$\begin{array}{c} C\;\left(t\right)=C_{0}\;\left[1-\exp\left(-\frac{t}{\tau }\right)\right] \end{array}$$

where *C*_0_ is essentially the maximum doping capacity of the polymer film and τ is the characteristic time for the reaction. (Note that τ is comparable but not identical to the switch time, which we always define as the time to reach 95% of the intensity change.) Combining Equations (2) and (3) gives

(4) $$\begin{array}{c} \log\left(R\right)=\log\left(R_{0}\right)-2C\sigma d\left[1-\exp\left(-\frac{t}{\tau }\right)\right] \end{array}$$

Thus, the logarithm of the reflected intensity should undergo a change characterized by an exponential dependence in time. This differs from previous studies which (without any physical basis) use an exponential fit directly for the intensity time trace.^[^ ^37^ ^]^ Note that our model does not consider any plasmonic activity being switched on and off,^[^ ^16^, ^38^ ^]^ assuming a simple reflective surface underneath the polymer (a constant *R*_0_). Therefore, we mainly used planar gold surfaces to evaluate the model accuracy, so that only the polymer contributes to the optical response. For the metasurfaces, one would need to introduce another time dependent term as *R*_0_ = *R*_0_((*C*(*t*)). However, the electrode geometry does not have to be considered because the model has no spatial dependence. (It is analogous to reaction‐limited surface binding.)

Our simple model provided excellent fits to the logarithmic intensity time trace (Figure 5b) when the switch speed was fast (≈50 ms or less). To the best of our knowledge, first order kinetics for the doping process has previously only been observed for polymers free in solution.^[^ ^39^ ^]^ We also confirmed that when the switch was considerably slower, the model no longer provided a good fit (Figure S13, Supporting Information), as expected when ion transport influences the rate. In this case, the model for the kinetics of the switch process needs to be extended, which is beyond the scope of the current work. However, we point out that such a model should not be based solely on ordinary diffusion (such as the Cottrell equation). We also emphasize the importance of using the optical readout when analyzing the kinetics of polaron formation rather than the current. First, the light intensity is obviously the key parameter for electrochromic devices. Second, the current contains contributions from processes other than polymer doping and accurate analysis is difficult.^[^ ^40^ ^]^ Indeed, we found that the integrated current during a switch did not scale linearly with the logarithm of the reflected intensity. The current is, however, essential for estimating the power consumption.

Video speed operation will lead to higher power consumption for any electronic paper technology since each switching process requires energy. However, we found that our devices still operated at low absolute powers. Examples of current time traces during “off” and “on” switching with ≈20 ms speed (Figure 5c) were used to estimate a maximum current density of 8.5 mA cm^−2^ during the switch (charge transfer less than 1.7 × 10^−4^ C cm^−2^). From the low voltages used, this translates to a power density of only 7 mW cm^−2^. This is considered as ultralow energy use and lies even below the typical value for organic LEDs.^[^ ^41^ ^]^ This is a huge overestimate as it also assumes full contrast switching very frequently. (Videos do not alternate between fully white and fully black frames at 50 Hz refresh rate.) Typical operation is likely to be around an order of magnitude lower,^[^ ^42^ ^]^ which gives an energy consumption less than 1 mW cm^−2^. In addition, our devices show bistability (Figure 5d). At open circuit potential we observed a slow drift approaching an equilibrium intensity level in ≈10 min. The bright or dark states of the polymer could easily be maintained by applying brief pulses. For the dark state, maintaining the intensity level so that it changed by less than 5% (of the total contrast) over time was possible by applying −0.7 V for 20 ms every ≈10 s. The bright state could be maintained with even less frequent pulsing (every ≈50 s). Furthermore, the current generated from these pulses was much lower than in Figure 5c, simply because the polymer doping state changed very little. Using the power densities measured during the pulses and considering that the power consumption is zero in between (open circuit), we calculated the energy cost for maintaining a steady image in a display device to be 0.23±0.05 µW cm^−2^ (average of dark and bright states). This value is orders of magnitude lower than both electrophoretic ink and bistable LCDs,^[^ ^43^ ^]^ mainly because those technologies require much higher voltages. This power consumption is also an overestimate since only a fraction of the pixels in a display would need to be fully bright or fully dark, i.e., many will be closer to the equilibrium doping state.

## 3 Conclusion

We have shown the first example of video speed (10–50 ms) and high contrast (Δ*R* ≈ 50%) switching of structural colors over the whole visible, which is a very important milestone for electronic paper technologies. More specifically, we have used plasmonic nanostructures and conductive polymers to avoid the use of polarizers. The metasurfaces generate a full color gamut superior to state‐of‐the‐art color e‐readers. Our high speed is the result of enhancing ion mobility together with the use of nanostructures that provide positive surface curvature. We emphasize that the electric field strength influences speed and the ion transport cannot be regarded as a purely diffusive process. Under fast‐switching conditions, the doping process perfectly follows first order reaction kinetics for polymer films sufficiently thick to provide high contrast. Remarkably, the device lifetime is increased to at least tens of millions of cycles because the fast switching only requires very brief potential pulses.

This work opens up unprecedented possibilities for electronic paper in color, with particular emphasis on the extended range of applications that come with video capabilities. At the same time, the ultralow power consumption will enable great power savings when replacing emissive displays in many scenarios. The next step is to implement the nanostructures on thin film transistor arrays for individual pixel addressing and polymer switching.^[^ ^44^ ^]^

## 4 Experimental Section

### Chemicals

Propylene carbonate, TBAClO_4_, BMIM, and acetonitrile were purchased from Sigma. LiClO_4_ was purchased from Fischer Scientific. The monomer was purchased from Sycon Polymers India and purified by making dispersion in deionized water (≈1 g monomer in 20 mL water) by sonication at 40 °C until a milky liquid was produced together with a brownish liquid. The milky liquid was transferred to another beaker, extracted, and recrystallized with hexane.

### Nanofabrication

Al and Al_2_O_3_ were deposited by physical vapor deposition (Lesker PVD 225) with electron gun heating. A 5 nm Cr layer was included under Al and a 1 nm Ti layer was included under Au to promote adhesion. The colloid adsorption step for generating a short‐range ordered pattern was performed as described previously.^[^ ^16^ ^]^ A polyelectrolyte layer was used to promote adhesion,^[^ ^16^ ^]^ and 54 nm polystyrene‐sulfate colloids (Invitrogen) were adsorbed from a pure water suspension until saturation. Samples were imaged by a Zeiss Supra 55 SEM. Topography was investigated in tapping mode with an NTEGRA AFM (NT‐MDT) with Tap300AI‐G tips (BudgetSensors) with resonant frequency of 200–400 kHz and force constant of 20–75 N m^−1^.

### Electrochemical Measurements

A home‐built liquid cell with Ag and Pt wires was used for three‐electrode measurements. The Ag wire was chloridized prior to experiments by applying anodic potentials^[^ ^24^ ^]^ (typically +1 V vs Pt) in 10 × diluted HCl in water. Lateral two‐electrode devices had 20 nm thin gold films as counter/reference electrode. A potentiostat (Gamry Interface 1000) was used for electropolymerization and switching. PProDOTMe_2_ was synthesized by linear voltage sweeps from −0.7 to +1.5 V at 200 mV s^−1^ in the presence of 0.1 m LiClO_4_ and 0.1 m of the monomer.^[^ ^13a^ ^]^ (Note that this was done in propylene carbonate for best uniformity, while almost all switching measurements were done in acetonitrile.) Impedance spectra were measured with 10 mV amplitude and −0.2 V DC bias using the Gamry Framework software.

### Optical Measurements

The chromaticity and reflectivity of the metasurfaces was analyzed in air and electrolyte using diffuse illumination by a CM‐700d spectrophotometer (Konika Minolta). The colors of the PocketBook were measured in the same manner, using the device presentation document included on this e‐reader, which shows circles containing the primary colors in different regions. The metasurfaces without polymer were measured while in contact with water since it differs in refractive index from the typical electrolyte environment by only 0.01. A custom microspectroscopy setup with beamsplitters (Thorlabs) was used to measure reflectivity on the microscale in the electrochemical cell.^[^ ^13a^ ^]^ The illumination (100 W tungsten lamp) and collection went through a 10× air objective (NA 0.25), looking through a cover glass window to the liquid cell. Part of the reflected light was collected by an optical fiber and analyzed by a spectrometer (B&WTek CypherX). In order to get the accurate absolute reflectivity, the mirror used to obtain reference intensity was also measured in the CM‐700d instrument as explained previously.^[^ ^13a^ ^]^

### Pictures

Flowers of different colors were picked by the wife of the corresponding author in their garden and a photo was taken with black background using a Huawei P30 Pro smartphone. Image processing into RGB values for reproduction was performed as in previous work.^[^ ^13b^ ^]^ The metasurfaces were prepared as RGB stripes of different length in three steps by laser lithography, using resists LOR3A and S1813 with developer MF‐318.^[^ ^13b^ ^]^ Microscopy images were taken with an Axiocam506 color camera in Zeiss Axio Observer 7 with a 50× air objective designed for both bright and dark field illumination in reflection mode. Other pictures and videos were taken with an Iphone11.

## Conflict of Interest

The authors declare no conflict of interest.

## Supporting information

Supporting Information

Supplemental Image

Supplemental Video 1

Supplemental Video 2

## Data Availability

The data that support the findings of this study are available from the corresponding author upon reasonable request.

## References

[1] K. Xiong , D. Tordera , M. P. Jonsson , A. B. Dahlin , Rep. Prog. Phys. 2019, 82, 024501. 30640724 10.1088/1361-6633/aaf844

[2] A. M. Chang , D. Aeschbach , J. F. Duffy , C. A. Czeisler , Proc. Natl. Acad. Sci. USA 2015, 112, 1232. 25535358 10.1073/pnas.1418490112 PMC4313820

[3] a) F. Neubrech , X. Duan , N. Liu , Sci. Adv. 2020, 6, eabc2709; 32917622 10.1126/sciadv.abc2709 PMC7473667

[4] J. Heikenfeld , P. Drzaic , J. S. Yeo , T. Koch , J. Soc. Inf. Disp. 2011, 19, 129.

[5] B. Comiskey , J. D. Albert , H. Yoshizawa , J. Jacobson , Nature 1998, 394, 253.

[6] M. Gugole , O. Olsson , S. Rossi , M. P. Jonsson , A. Dahlin , Nano Lett. 2021, 21, 4343. 33969987 10.1021/acs.nanolett.1c00904 PMC8289301

[7] R. A. Hayes , B. J. Feenstra , Nature 2003, 425, 383. 14508484 10.1038/nature01988

[8] D. Franklin , Z. He , P. Mastranzo Ortega , A. Safaei , P. Cencillo‐Abad , S.‐T. Wu , D. Chanda , Proc. Natl. Acad. Sci. USA 2020, 117, 13350. 32493745 10.1073/pnas.2001435117 PMC7306820

[9] V. Rai , R. S. Singh , D. J. Blackwood , D. Zhili , Adv. Eng. Mater. 2020, 22, 2000082.

[10] J. Heinze , B. A. Frontana‐Uribe , S. Ludwigs , Chem. Rev. 2010, 110, 4724. 20557047 10.1021/cr900226k

[11] J. C. Lacroix , K. K. Kanazawa , A. Diaz , J. Electrochem. Soc. 1989, 136, 1308.

[12] a) V. Jain , H. M. Yochum , R. Montazami , J. R. Heflin , Appl. Phys. Lett. 2008, 92, 033304;

[13] a) M. Gugole , O. Olsson , K. Xiong , J. C. Blake , J. Montero Amenedo , I. Bayrak Pehlivan , G. A. Niklasson , A. Dahlin , ACS Photonics 2020, 7, 1762;

[14] M. Kateb , S. Safarian , M. Kolahdouz , M. Fathipour , V. Ahamdi , Sol. Energy Mater. Sol. Cells 2016, 145, 200.

[15] M. Shahabuddin , T. McDowell , C. E. Bonner , N. Noginova , ACS Appl. Nano Mater. 2019, 2, 1713.

[16] K. Xiong , G. Emilsson , A. Maziz , X. Yang , L. Shao , E. W. H. Jager , A. B. Dahlin , Adv. Mater. 2016, 28, 9956. 27670834 10.1002/adma.201603358

[17] H. Takei , M. Himmelhaus , T. Okamoto , Opt. Lett. 2002, 27, 342. 18007797 10.1364/ol.27.000342

[18] T. A. Kelf , Y. Sugawara , R. M. Cole , J. J. Baumberg , M. E. Abdelsalam , S. Cintra , S. Mahajan , A. E. Russell , P. N. Bartlett , Phys. Rev. B 2006, 74, 245415.

[19] a) A. D. Rakic , Appl. Opt. 1995, 34, 4755; 21052313 10.1364/AO.34.004755

[20] P. K. Jain , K. S. Lee , I. H. El‐Sayed , M. A. El‐Sayed , J. Phys. Chem. B 2006, 110, 7238. 16599493 10.1021/jp057170o

[21] D. M. Welsh , A. Kumar , E. W. Meijer , J. R. Reynolds , Adv. Mater. 1999, 11, 1379.

[22] a) T. Xu , E. C. Walter , A. Agrawal , C. Bohn , J. Velmurugan , W. Zhu , H. J. Lezec , A. A. Talin , Nat. Commun. 2016, 7, 10479; 26814453 10.1038/ncomms10479 PMC4737852

[23] R. Brooke , E. Mitraka , S. Sardar , M. Sandberg , A. Sawatdee , M. Berggren , X. Crispin , M. P. Jonsson , J. Mater. Chem. C 2017, 5, 5824.

[24] A. B. Dahlin , R. Zahn , J. Voros , Nanoscale 2012, 4, 2339. 22374047 10.1039/c2nr11950a

[25] J. J. Vos , Color Res. Appl. 1978, 3, 125.

[26] T. Johansson , N. K. Persson , O. Inganas , J. Electrochem. Soc. 2004, 151, E119.

[27] E. Stavrinidou , P. Leleux , H. Rajaona , D. Khodagholy , J. Rivnay , M. Lindau , S. Sanaur , G. G. Malliaras , Adv. Mater. 2013, 25, 4488. 23784809 10.1002/adma.201301240

[28] A. Kumar , D. M. Welsh , M. C. Morvant , F. Piroux , K. A. Abboud , J. R. Reynolds , Chem. Mater. 1998, 10, 896.

[29] E. Y. Tyunina , M. D. Chekunova , Russ. J. Phys. Chem. A 2017, 91, 894.

[30] T. F. Otero , I. Boyano , J. Phys. Chem. B 2003, 107, 6730.

[31] S. I. Cho , S. B. Lee , Acc. Chem. Res. 2008, 41, 699. 18505276 10.1021/ar7002094

[32] X. Wang , B. Shapiro , E. Smela , Adv. Mater. 2004, 16, 1605.

[33] D. Tu , S. Fabiano , Appl. Phys. Lett. 2020, 117, 080501.

[34] a) W. Lu , A. G. Fadeev , B. H. Qi , B. R. Mattes , J. Electrochem. Soc. 2004, 151, H33;

[35] E. Stavrinidou , P. Leleux , H. Rajaona , M. Fiocchi , S. Sanaur , G. G. Malliaras , J. Appl. Phys. 2013, 113, 244501.

[36] I. Sahalianov , S. K. Singh , K. Tybrandt , M. Berggren , I. Zozoulenko , RSC Adv. 2019, 9, 42498. 35542835 10.1039/c9ra10250g PMC9076818

[37] S. Hassab , D. E. Shen , A. M. Osterholm , M. Da Rocha , G. Song , Y. Alesanco , A. Vinuales , A. Rougier , J. R. Reynolds , J. Padilla , Sol. Energy Mater. Sol. Cells 2018, 185, 54.

[38] J. Ratzsch , J. Karst , J. Fu , M. Ubl , T. Pohl , F. Sterl , C. Malacrida , M. Wieland , B. Reineke , T. Zentgraf , S. Ludwigs , M. Hentschel , H. Giessen , J. Opt. 2020, 22, 124001.

[39] F. M. McFarland , L. R. Bonnette , E. A. Acres , S. Guo , J. Mater. Chem. C 2017, 5, 5764. 10.1039/C7TC00189D PMC5646835 29057077

[40] A. M. Osterholm , J. F. Ponder , M. De Keersmaecker , D. E. Shen , J. R. Reynolds , Chem. Mater. 2019, 31, 2971.

[41] M. R. Fernandez , E. Z. Casanova , I. G. Alonso , Sustainability 2015, 7, 10854.

[42] S. V. Porter , M. Mirmehdi , B. T. Thomas , Proc. 15th Int. Conf. on Pattern Recognition. ICPR‐2000, Vol. 3, IEEE, Piscataway, NJ, USA 2000, pp. 409–412, 10.1109/ICPR.2000.903571.

[43] J. C. Jones , J. Soc. Inf. Disp. 2008, 16, 143.

[44] B. Bao , B. Rivkin , F. Akbar , D. D. Karnaushenko , V. K. Bandari , L. Teuerle , C. Becker , S. Baunack , D. Karnaushenko , O. G. Schmidt , Adv. Mater. 2021, 33, 2101272. 10.1002/adma.202101272 PMC11469128 34028906

