# Supplementary material for: Video Speed Switching of Plasmonic Structural Colors with High Contrast and Superior Lifetime
Source: Adv Mater. 2021 Aug 26;33(41):2103217. doi: 10.1002/adma.202103217 (PMC11468514; doi:10.1002/adma.202103217)
Supplement: Supplementary file 1 — Supporting Information [file ADMA-33-2103217-s002.pdf]

© 2021 Wiley-VCH GmbH

ADVANCED MATERIALS

Supporting Information

for *Adv. Mater.,* DOI: 10.1002/adma.202103217

Video Speed Switching of Plasmonic Structural Colors with High Contrast and Superior Lifetime

*Kunli Xiong, Oliver Olsson, Justas Svirelis, Chonnipa Palasingh, Jeremy Baumberg, and Andreas Dahlin**

Numerical simulations of optical properties

Numerical simulations were performed in COMSOL Multiphysics 5.0 using the Wave Optics module and frequency domain. Literature data was used for permittivity of Al and Au (see references in main text). The dielectrics were modelled as non-dispersive with refractive index 1.73 for Al_2O_3 and 1.53 for polystyrene, while 1.4 was used for glass and electrolyte. Periodic boundary conditions were used (square lattice) and the periodicity was set to 155 nm. The long-range ordered array in the simulation accurately describes the optical response from the short-range ordered samples for these characteristic spacings (see cited references).

The exact geometry of particles and metal layers are shown in the near field plots. The simulated spectra (Figure S1) are clearly in good agreement with the experimental (main text). The near fields show that a plane wave is reflected at the reflectivity maxima. In spectral regions with low reflectivity, the influence from localized resonances is clear. For blue and green, this is a "shell" resonance (field enhanced in the void), while it is closer to a "particle" resonance for the red samples (field enhanced at the protrusion). There is no strong field enhancement at the buried colloid for the red samples.

Note that the simulations assume perfect size homogeneity for the colloids. This is quite far from the real samples, but as mentioned the spectra are still very similar to the experimental ones. One likely explanation why the resonances are broadened overall is plasmonic coupling. This may occur directly between neighboring particles but also through the continuous metal film, which allows coupling to propagating modes as a decay channel for the localized modes.

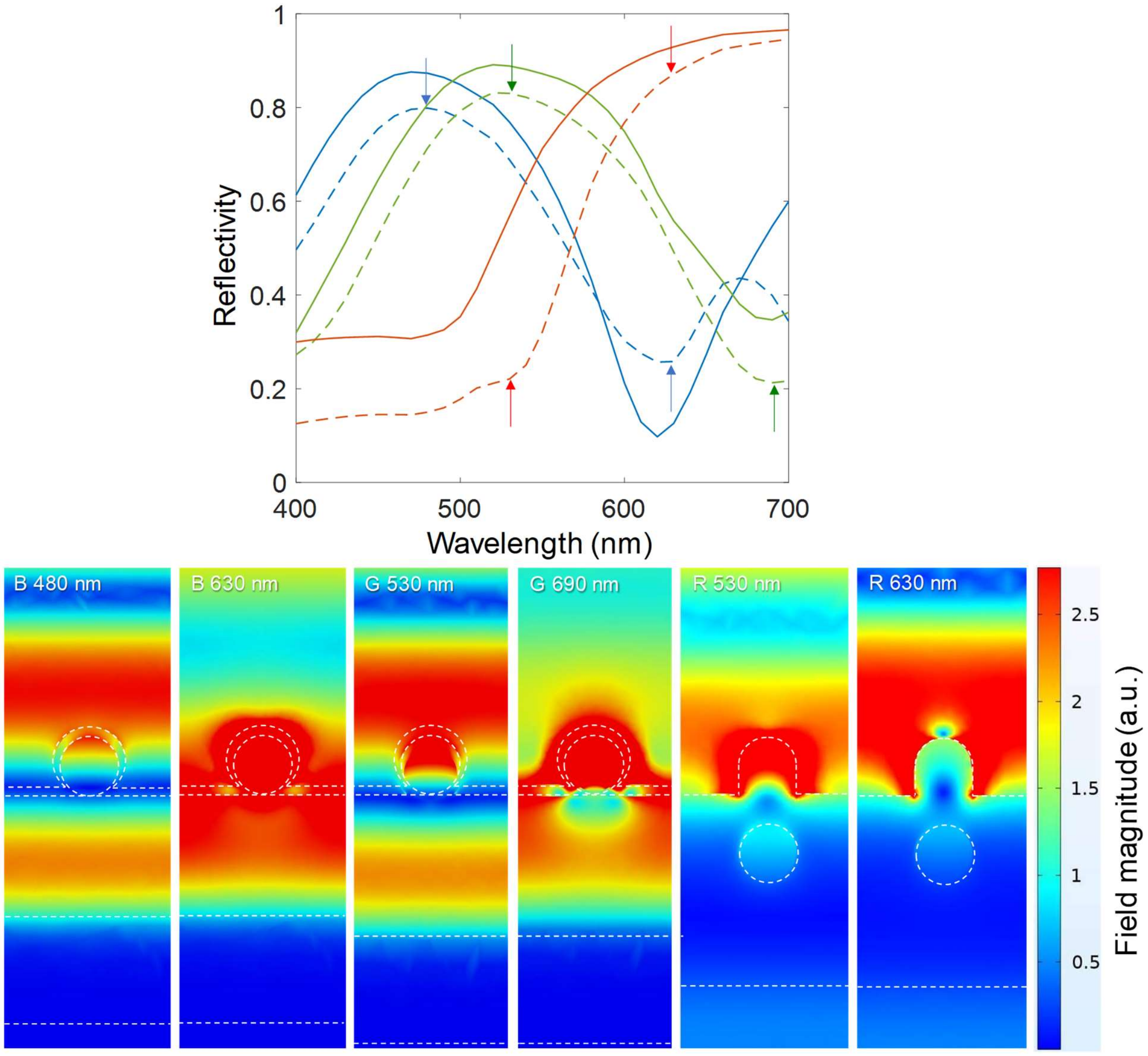

Figure S1. Simulated reflectivity for red, green and blue metasurfaces. The arrows indicate the wavelengths where the near fields are visualized. White dashed lines show the boundaries.

Other colors

As expected, by fine tuning structural parameters such as particle size, materials and thickness of the deposited films, many colors can be produced by our metasurface design approach. With further work it may even be possible to improve the primary RGB colors, but this paper focuses on the video speed operation of the system. An example of different colors is shown in Figure S2. For instance, cyan and yellow is clearly achievable. It is also shown that the structures can be bent, i.e. it is possible to make foldable display devices.

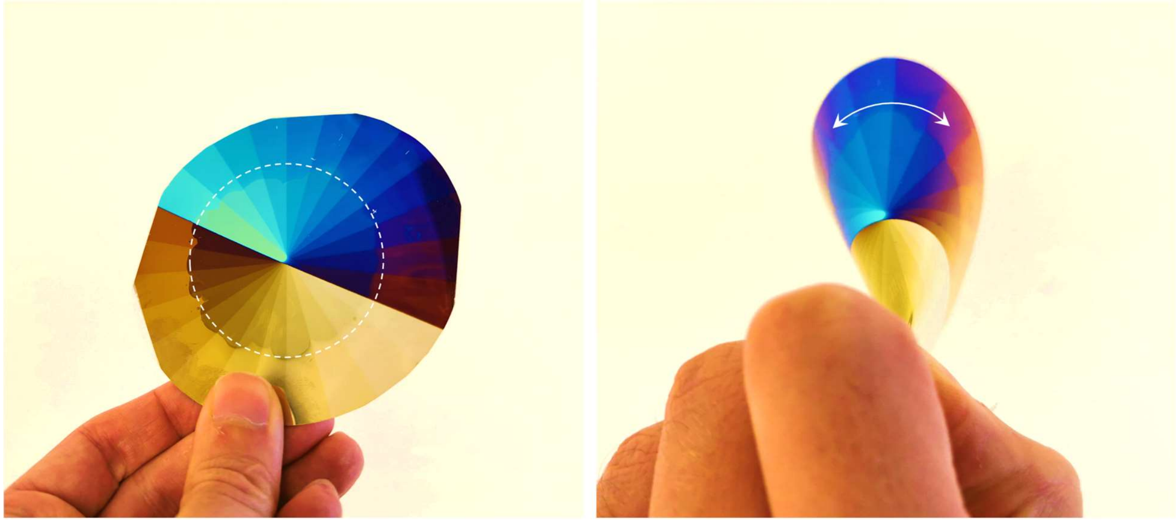

Figure S2. Example of color tuning by changing Al_2O_3 thickness. The middle part of the sample has particles. The plastic support is flexible and the structure can be bent without damage.

Dark field spectra

Scattering spectra are shown in Figure S3. The light source reference spectrum was measured using a white diffuser. The red samples showed stronger scattering than blue and green.

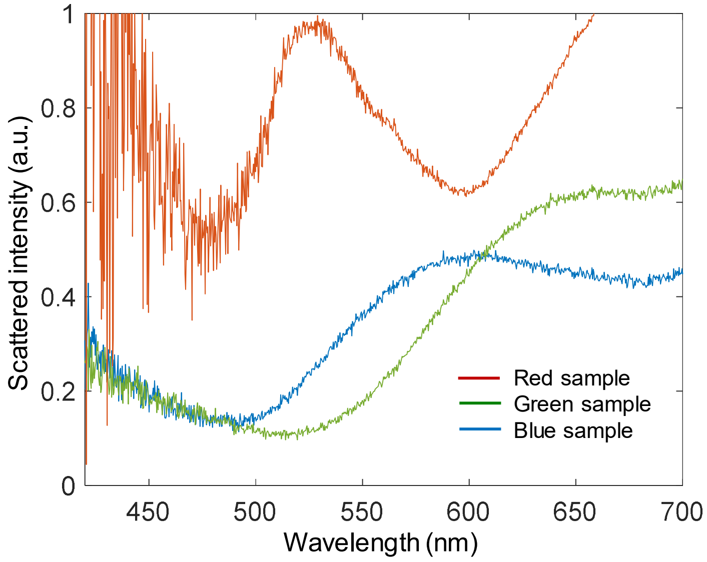

Figure S3. Scattering spectra measured in air.

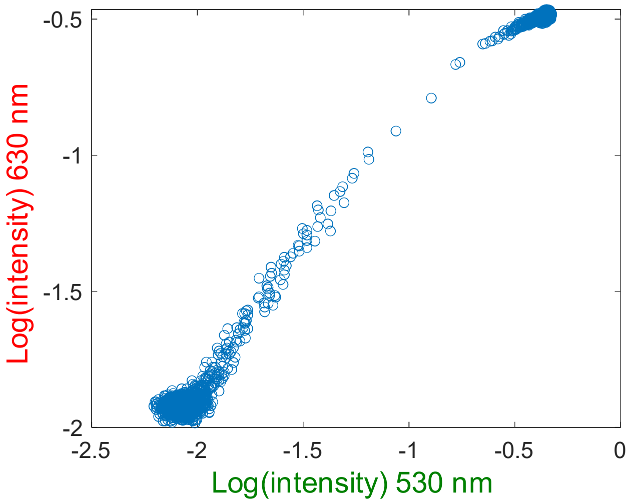

Figure S4. Intensities at two different wavelengths during a switch. Since the relation is close to linear it matters very little which wavelength is chosen when it comes to the final values of τ and the quality of the fit. The small deviation from linearity may be due to bipolaron formation at higher oxidation states, since these entities will not have the same absorption characteristics as the polarons that are initially formed.

Imaging the switching process in lateral devices

Although a vertical configuration is preferable in a real display device, the lateral devices provided much insight into what influences the switching speed. By imaging with a camera (Andor iXon Life CCD) we observed that the speed was faster closer to the electrode edge (regardless of electrode separation distance). Our interpretation is that there is no significant voltage drop along the metallic electrode and that it is the enhanced field closer to the edge which makes the ions move faster. The data in Figure S5 shows that the switch is faster closer to the edge, suggesting that the higher field also extracts ions faster from the polymer. Note that this shows the off switch, which is faster than the on switch, but still dependent on the field. (The switching front behavior was the same qualitatively whether the switch was "on" or "off".) Note also that although the camera provides slightly higher temporal resolution than the spectrometer, the intensity values were found to be noisier, which is why they were not used for quantitative analysis.

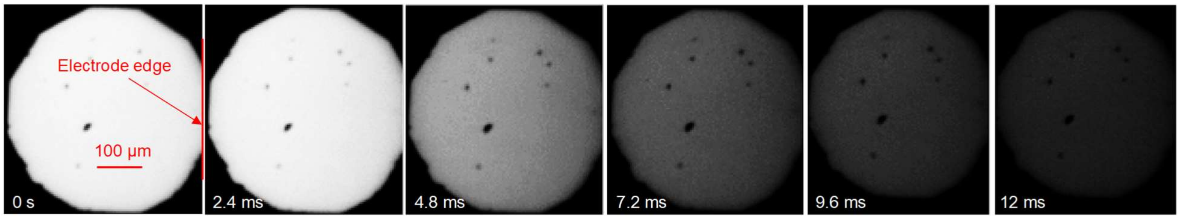

Figure S5. High speed camera imaging of a moving "switching front" in a lateral device. The edge of the analyzed electrode is just at the right edge of each image and the counter electrode is 100 μm away to the right. During switching, the speed is clearly faster closer to the edge.

Simulations of field in lateral and vertical devices

The field between lateral electrodes was simulated by electrostatics in COMSOL Multiphysics (Figure S6). The potential does not change along the gold surface due to its high conductivity compared to the electrolyte. For comparison, two parallel electrodes with 100 μm separation will generate a homogenous field with a magnitude equal to 10^4 Vm^{-1}. Such high values are only reached just at the edges in the lateral configuration, which confirms that the vertical configuration is preferable for promoting ion movement (but less convenient for a systematic investigation of effects from electrode separation distance and measurement position). Once the potential is applied, the field will naturally change quickly due to electrochemical processes, but the simulations can at least be used to represent the initial distribution and make a relative comparison of field strengths with different electrode geometries.

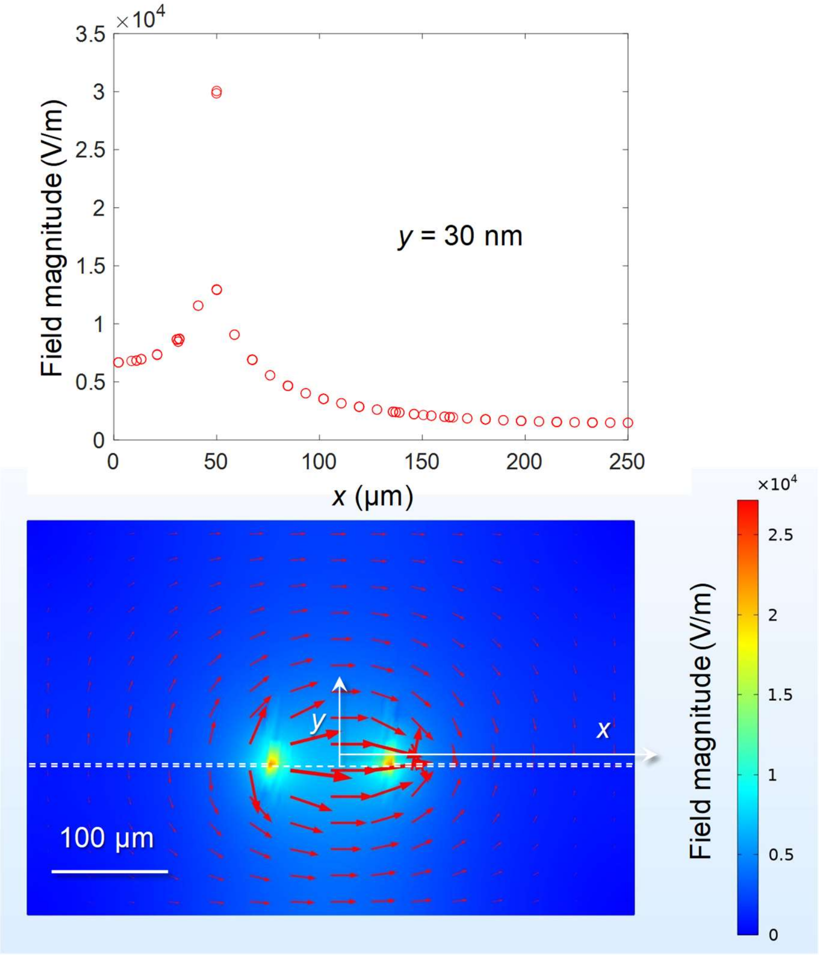

Figure S6. Electrostatic field distribution for 20 nm gold electrodes with lateral separation of 100 µm. The potential difference is 1 V. The DC permittivity is 2 for glass and 1 for the medium. (The purpose is not to accurately represent the electrolyte, only to give the initial field distribution and compare positions along the electrode.)

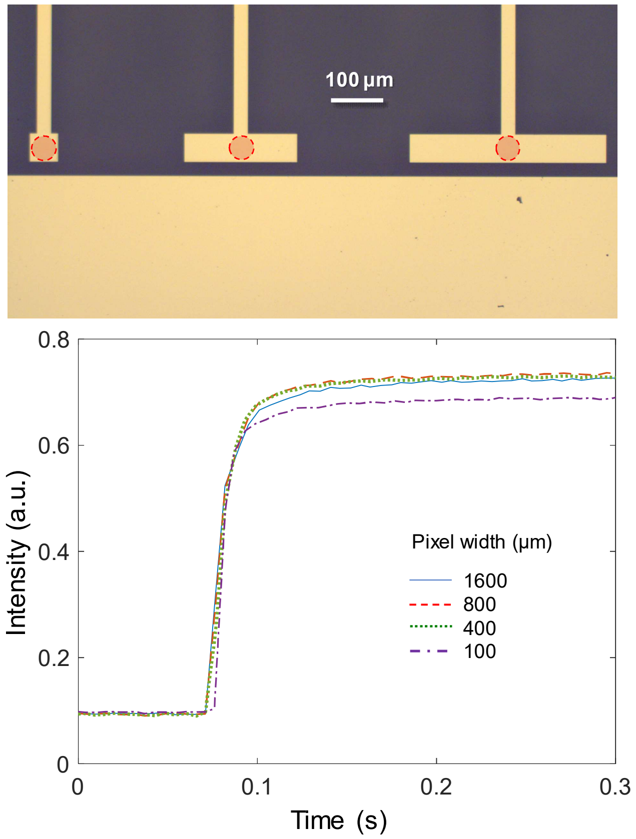

Figure S7. Example of a switch speed test on pixels of different size and geometries. In this case, the width was varied while maintaining the same distance to the counter electrode. No significant difference in switch speed could be detected. The top image shows some of the pixels with the measurement spots indicated.

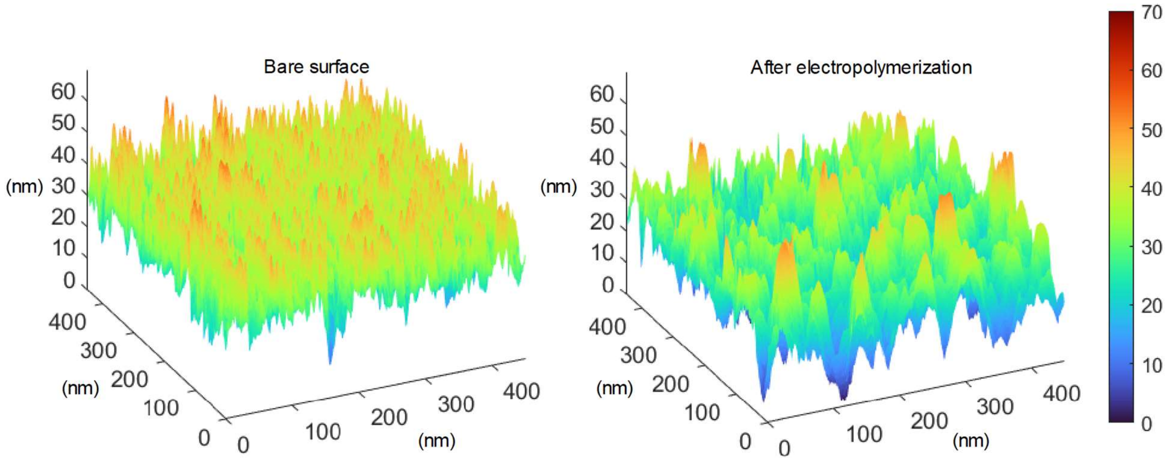

Figure S8. Atomic force microscopy images showing a nanostructure before and after polymerization. The sample changes, in particular with respect to the magnitude of height variations (RMS roughness increased). This suggests that the polymer deposits primarily on the protrusions. (A conformal coating should not lead to any changes and polymer growth only on the planar regions would decrease the height variation.) Data was analyzed in Gwyddion. For both images, plane fit and row alignment was performed in the same manner.

Curvature dependence

Figure S9 shows switch speeds measured on planar surfaces and nanostructures prepared like the red samples but with larger colloids (150 nm). Due to changes in plasmonic properties, these structures did not have a red color, but the reflected intensity can of course still be tracked during switching and switch times extracted (as they are based on relative intensity changes). The switch time clearly fall in between those measured on the metasurfaces and those measured on planar gold. This shows that higher curvature promotes faster switching (for the same total polymer amount), although more experiments are needed to establish quantitative relations.

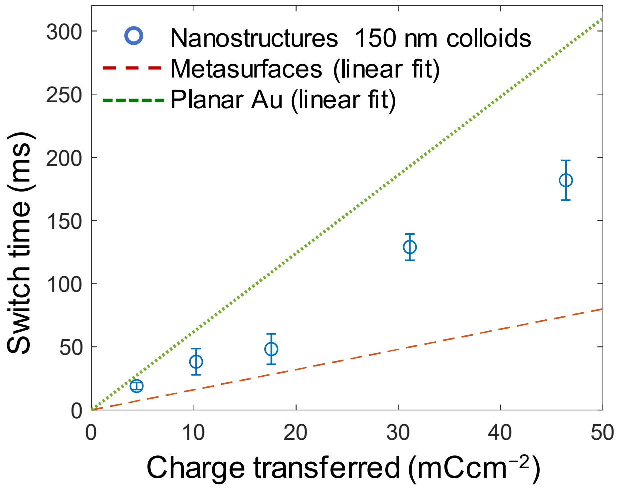

Figure S9. Switch speeds measured on samples with less curvature. The lines show the fits to data shown in the main text. The "charge transferred" is measured during polymerization and proportional to the amount of polymer on the surfaces.

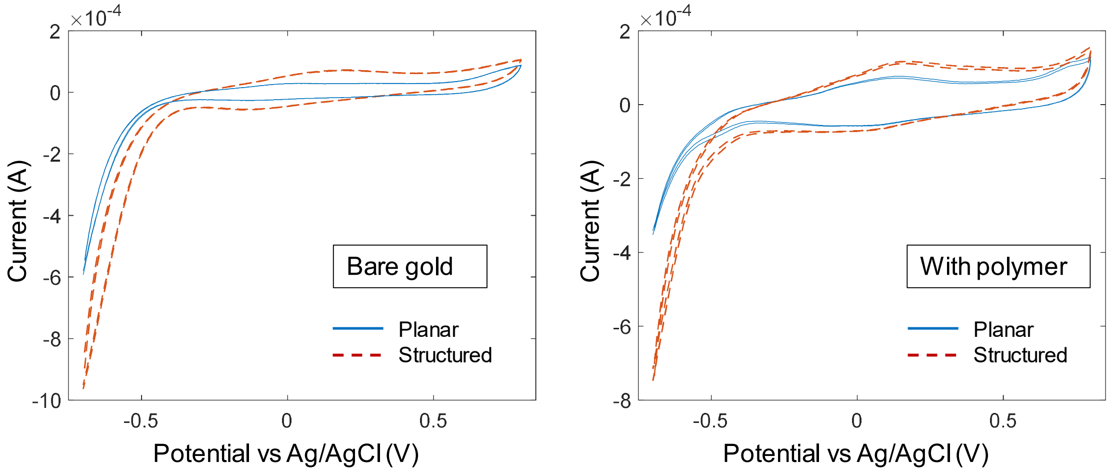

Figure S10. Cyclic voltammetry (1 M $LiClO_4$ in acetonitrile, 100 mVs^{-1}) of empty surfaces and after electropolymerization (planar gold or red metasurface). The polymer amount is similar on both surfaces (12±1 $mCcm^{-2}$). The polymer redox activity starts to appear around 0 V vs Ag/AgCl. (Current peaks become more pronounced for thicker films.) Two cycles are shown in each case. The electrode area was 1.76 mm^2. The current generated at negative voltage clearly has nothing to with the polymer as it appears on the unmodified gold. It can be attributed to water splitting since the solvent always takes up a small amount of water from ambient air. The metasurface has a slightly higher current, which corresponds well to its higher effective area due to the protrusions.

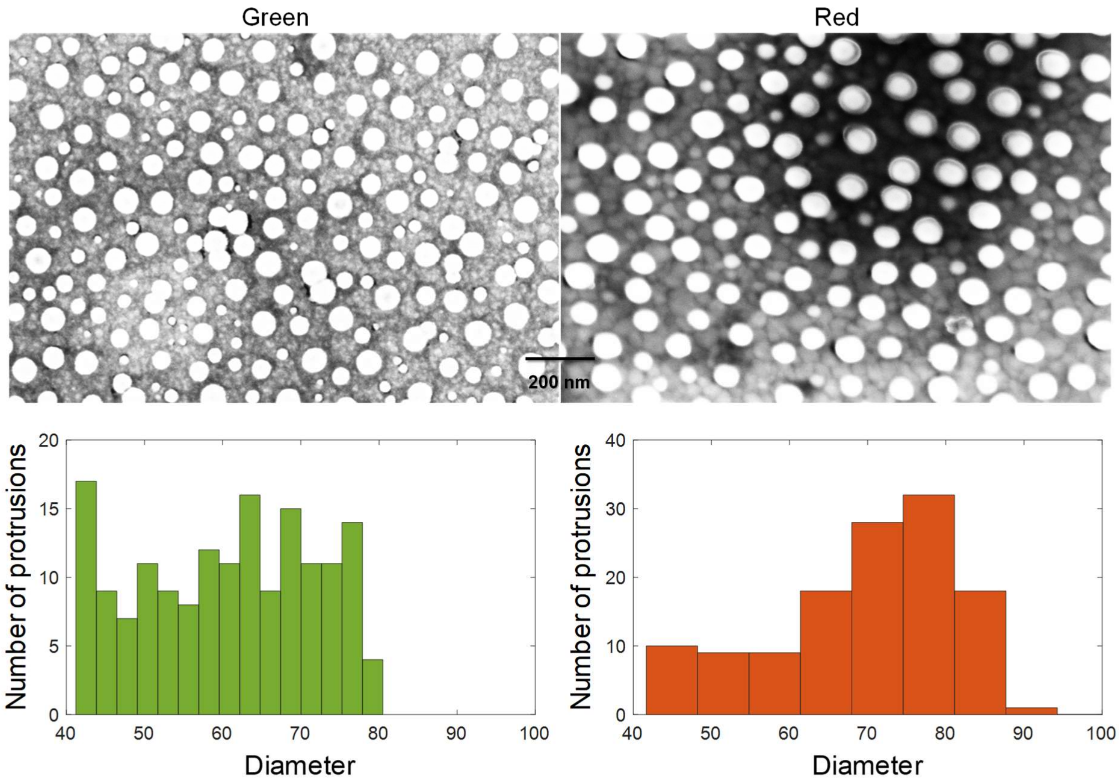

Figure S11. SEM image analysis of a green and a red sample. The red sample has slightly higher diameter protrusions on average and a narrower distribution. This is most likely because less metal is deposited on the green (and blue) samples and the tilt during the deposition leads to shadowing effects. Note that increasing the colloid amount is possible by including salt (instead of pure water) to screen their repulsion during adsorption, but this leads to many connected protrusions.

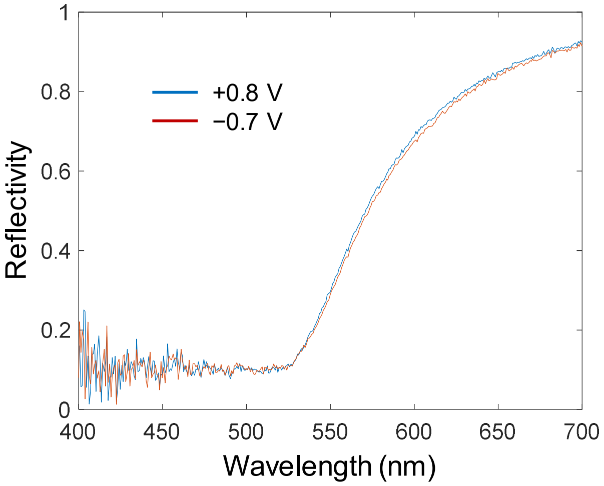

Figure S12. Switching a (red) sample without polymer as a control experiment (1 M $LiClO_4$ in acetonitrile). Spectral changes are detectable but very small.

Kinetic model fit for thick films

For thicker and thicker films with slower and slower switching, the single exponential model was less and less accurate for describing the data. Some examples are shown in Figure S13. This confirms that ion transport still influences the switch speed for sufficiently thick films, even though measures are taken to enhance the drift motion. The same poor fitting can be achieved by using long electrode separation distance or high viscosity solvents.

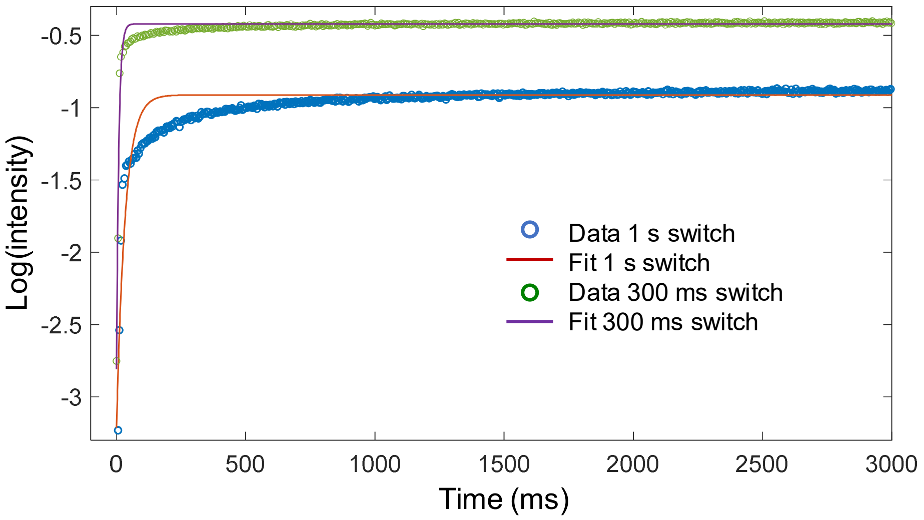

Figure S13. Best fits to first order reaction kinetics for slower "on" switches. Time to reach 95% of the intensity change is either 300 ms (R^2 = 0.939) or 1 s (R^2 = 0.813). Note that the y-axis shows the logarithm of the reflected intensity.

We note that although our particular system had its switch speed enhanced by promoting drift motion and approached a "reaction limited" regime, we cannot know that other polymer/electrolyte combinations will behave similarly. If the ion movement process would be purely diffusive, the switch time is expected to scale with the square of the film thickness. This relation is often mentioned in existing literature (see discussion in main text) but we are not aware of any report showing experimental data that clearly supports such a dependence.

Image S1 (separate file)

Image used for color photo reproduction in jpg format.

Video S1 (separate file)

Viewing angle dependence. A blue and a red sample are rotated. (Both are covered with a thin layer of liquid and a glass slide.)

Video S2 (separate file)

Example of switching at different speeds.
