# Supplementary figures and images for: Video Speed Switching of Plasmonic Structural Colors with High Contrast and Superior Lifetime
Source: Adv Mater. 2021 Aug 26;33(41):2103217. doi: 10.1002/adma.202103217 (PMC11468514; doi:10.1002/adma.202103217)

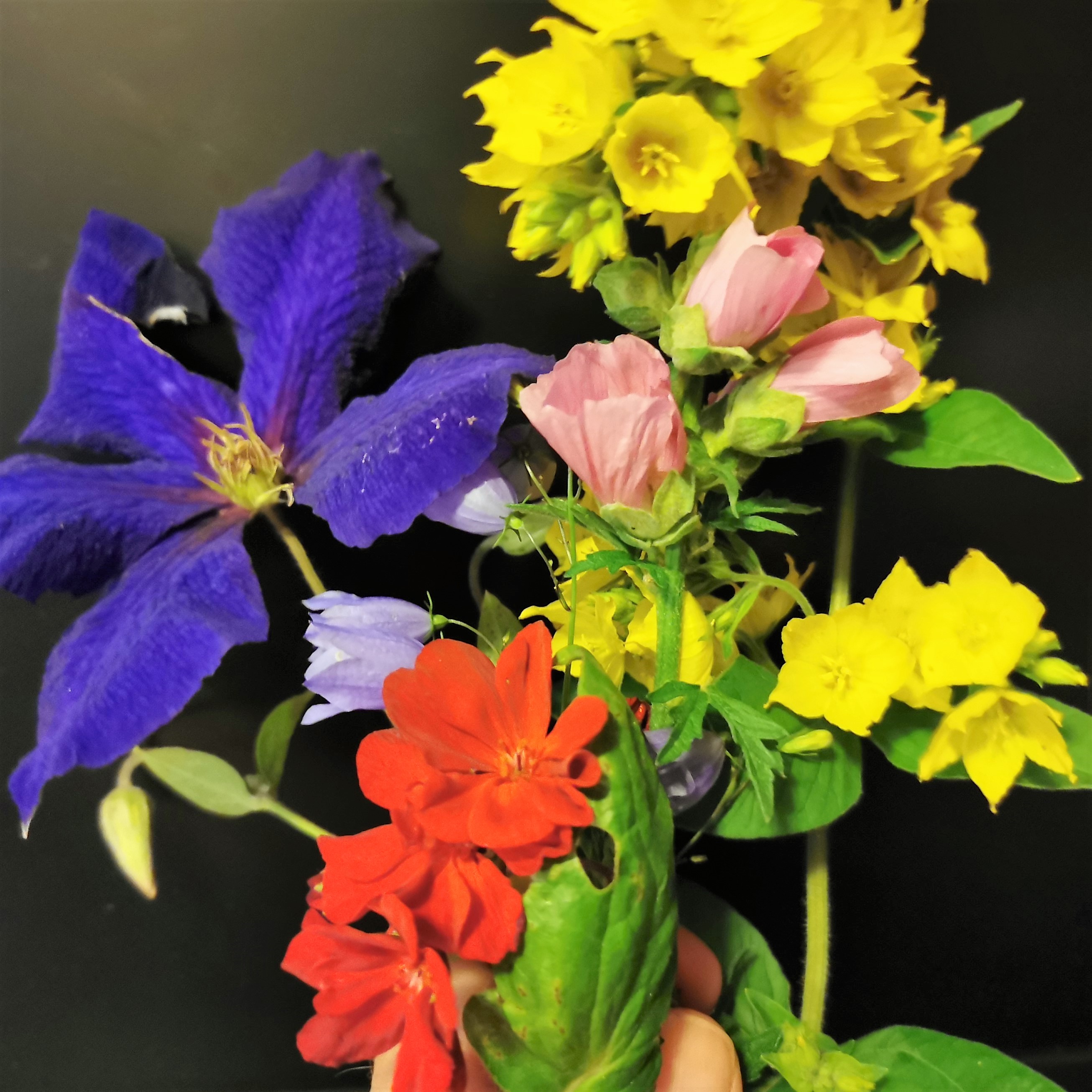

Supplement: Supplementary file 2 — Supplemental Image [file ADMA-33-2103217-s004.jpg]
